# A Unique Role of the Human Cytomegalovirus Small Capsid Protein in Capsid Assembly

**DOI:** 10.1128/mbio.01007-22

**Published:** 2022-09-06

**Authors:** Eva Maria Borst, Sarah Harmening, Saskia Sanders, Enrico Caragliano, Karen Wagner, Tihana Lenac Roviš, Stipan Jonjić, Jens Bernhard Bosse, Martin Messerle

**Affiliations:** a Department of Virology, Hannover Medical School, Hannover, Germany; b Leibniz-Institute for Experimental Virology, Hamburg, Germany; c Centre for Structural Systems Biology, Hamburg, Germany; d Cluster of Excellence RESIST (EXC 2155), Hannover Medical School, Hannover, Germany; e Center for Proteomics, Faculty of Medicine, University of Rijeka, Rijeka, Croatia; f German Center for Infection Research, Partner Site Hannover Braunschweig, Hannover, Germany; University of North Carolina, Chapel Hill

**Keywords:** capsid assembly, cytomegalovirus, genome packaging, small capsid protein

## Abstract

Morphogenesis of herpesvirus particles is highly conserved; however, the capsid assembly and genome packaging of human cytomegalovirus (HCMV) exhibit unique features. Examples of these include the essential role of the small capsid protein (SCP) and the existence of the β-herpesvirus-specific capsid-associated protein pp150. SCP and pp150, as well as the UL77 and UL93 proteins, are important capsid constituents, yet their precise mechanism of action is elusive. Here, we analyzed how deletion of the open reading frames (ORFs) encoding pUL77, pUL93, pp150, or SCP affects the protein composition of nuclear capsids. This was achieved by generating HCMV genomes lacking the respective genes, combined with a highly efficient transfection technique that allowed us to directly analyze these mutants in transfected cells. While no obvious effects were observed when pUL77, pUL93, or pp150 was missing, the absence of SCP impeded capsid assembly due to strongly reduced amounts of major capsid protein (MCP). Vice versa, when MCP was lacking, SCP became undetectable, indicating a mutual dependence of SCP and MCP for establishing appropriate protein levels. The SCP domain mediating stable MCP levels could be narrowed down to a C-terminal helix known to convey MCP binding. Interestingly, an SCP-EGFP (enhanced green fluorescent protein) fusion protein which also impaired the production of infectious progeny acted in a different manner, as capsid assembly was not abolished; however, SCP-EGFP-harboring capsids were devoid of DNA and trapped in paracrystalline nuclear structures. These results indicate that SCP is essential in HCMV because of its impact on MCP levels and reveal SCP as a potential target for antiviral inhibitors.

## INTRODUCTION

Infection with human cytomegalovirus (HCMV) is ubiquitous in the human population, with seroprevalences reaching up to 100% (e.g., in developing countries). Although mainly asymptomatic in healthy individuals, HCMV is a major risk factor for immunocompromised and immunologically immature individuals. Accordingly, HCMV often leads to life-threatening complications in transplant patients, and moreover, is the most common viral cause of birth defects such as mental retardation and deafness (1).

**FIG 1:**
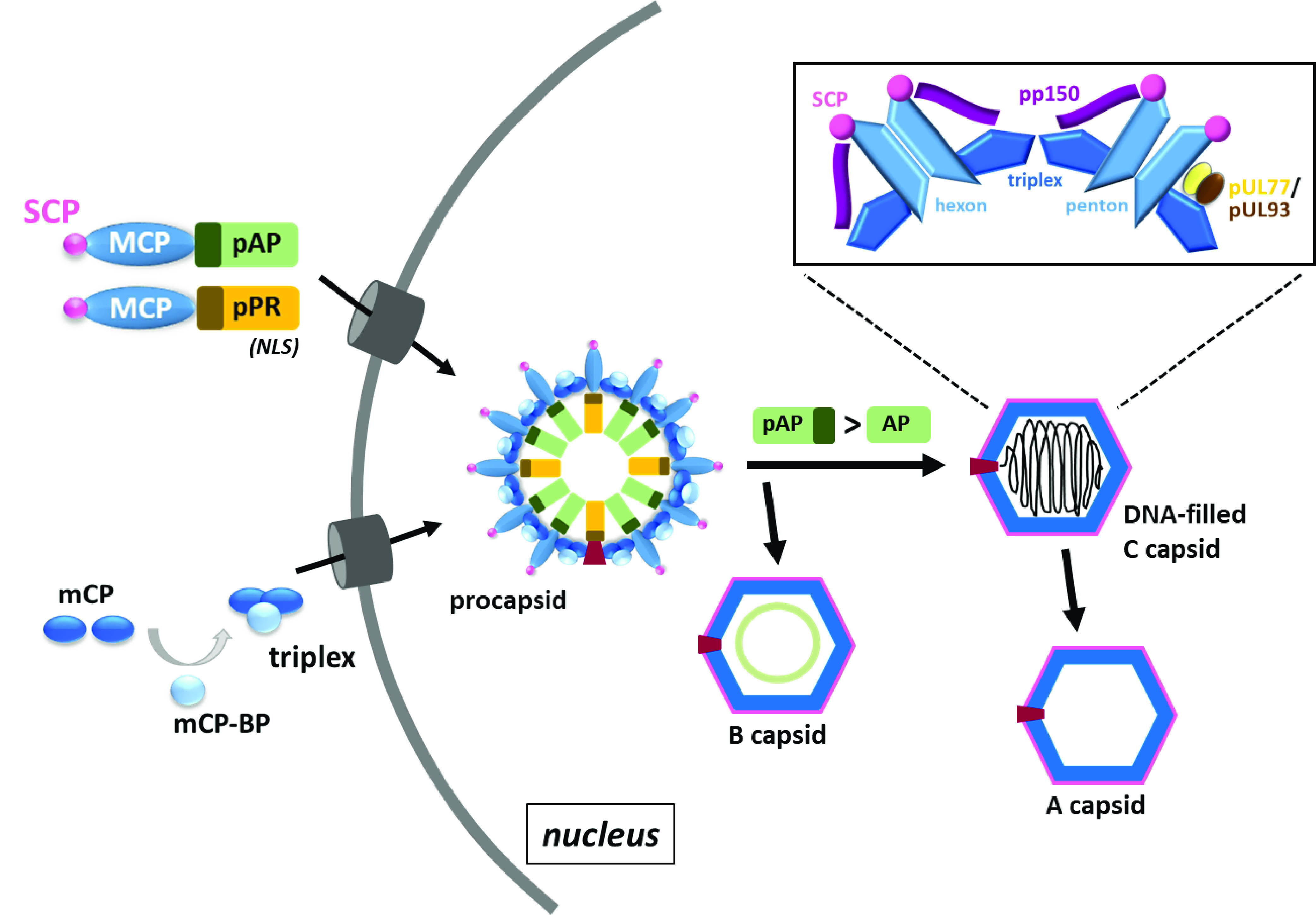
Schematic overview of human cytomegalovirus (HCMV) capsid assembly steps. In the cytoplasm, major capsid protein (MCP) interacts with both small capsid protein (SCP) and scaffolding proteins (assembly protein precursor [pAP] and protease precursor [pPR]), the latter providing a nuclear localization signal (NLS). Likewise, the triplex comprising two copies of the minor capsid protein (mCP) and one copy of the minor capsid protein-binding protein (mCP-BP) is also formed in the cytoplasm. Upon nuclear import, procapsids are assembled which, following genome encapsidation, mature into DNA-filled C capsids. Scaffold cleavage (pAP → AP) occurs simultaneously with genome packaging and capsid angularization. Loss of viral DNA due to incomplete genome encapsidation or failure to stabilize the DNA-filled capsids results in empty A capsids, and spontaneous angularization of procapsids yields B capsids, which retain the scaffold but do not contain viral DNA. Inset panel: arrangement of SCP and capsid-associated proteins pp150, pUL77, and pUL93. SCP and pp150 are in close contact with hexons and pentons, and in roughly half of the capsids (derived from extracellular virions), a few pp150 molecules at pentons are replaced by the capsid vertex-specific complex proteins pUL77/pUL93.

HCMV belongs to the herpesviruses and is the prototype member of the Betaher-pesvirinae subfamily. Morphogenesis of HCMV particles proceeds similarly to other herpesviruses, although there are some important differences. HCMV capsids are icosahedral structures in which the major capsid protein (MCP) is arranged into 150 hexons and 11 penton vertices. The 12th vertex is built by the dodecameric portal through which the viral genome is translocated into the capsid. Hexons and pentons are linked at the capsid floor by the triplex proteins made of two copies of the minor capsid protein (mCP) and one copy of the mCP-binding protein (mCP-BP). The small capsid protein (SCP) decorates the tips of MCP molecules and is the most divergent capsid protein within the *Herpesviridae*, both structurally and functionally (1, 2). Notably, SCP is essential in β-herpesviruses (3), while in α- and γ-herpesviruses it is not (4, 5). HCMV capsid assembly begins in the cytoplasm with interaction between the small and major capsid protein which in turn binds to the scaffolding proteins pAP (assembly protein precursor) and pPR (protease precursor; Fig. 1). Likewise, triplex proteins mCP and mCP-BP also associate in the cytoplasm. Following nuclear importation of the capsid and scaffolding proteins (mediated by nuclear localization signals in the scaffold and mCP-BP), spherical procapsids are assembled, which are thought to be the substrate for DNA packaging (6–8). The 240-kbp linear HCMV DNA genome is replicated as a concatemer of head-to-tail-linked viral genomes, whose encapsidation shares similarities with those of tailed bacteriophages, involving DNA-metabolizing enzymes named terminases that cleave the concatemers into unit-length genomes. Successful genome packaging occurs concurrently with activation of pPR by a yet-unknown mechanism, followed by cleavage of pAP into AP and expulsion of the scaffold cleavage products (Fig. 1). The resulting DNA-filled C capsids undergo nuclear egress, and subsequent assembly of infectious progeny in the cytoplasm includes the addition of tegument proteins and envelopment with a lipid membrane. Abortive DNA-packaging events result in empty shells, termed A capsids, and spontaneous angularization of procapsids due to untimely scaffold cleavage gives rise to B capsids which retain the scaffold but lack the viral DNA genome (1). Both A and B capsids are considered dead-end products of viral genome packaging (Fig. 1), although intermediate forms of B capsids are also reported to engage in genome encapsidation (8–10).

Besides capsid and terminase components, additional essential viral proteins are necessary for HCMV DNA encapsidation. These include pUL77 and pUL93, which are constituents of nuclear capsids. Both proteins are required for the generation of DNA-filled capsids, as no cleavage of viral concatemers and only empty B capsids were found upon deletion of the UL77 or UL93 open reading frames (ORFs), as shown by pulsed-field gelelectrophoresis (for the UL77 and UL93 null genomes) and analysis of free genomic ends (for a UL93 stop mutant [11, 12]). Notably, the phenotype of the HCMV-ΔUL77 mutant is distinct from those of corresponding α-herpesvirus mutants (herpes simplex virus 1 [HSV-1] or pseudorabiesvirus [PRV]), inasmuch as unit-length genomes and C capsids are obtained with HSV-1 and PRV mutants deleted for the orthologous UL25 gene (13–15). This suggests a different role of pUL77 compared to its UL25 ortholog in α-herpesviruses. In PRV and HSV-1, pUL25 and pUL17 (the HCMV UL93 ortholog) build the capsid vertex-specific complex (CVSC) located at the penton vertices, stabilizing C capsids after genome packaging (16, 17). Moreover, pUL25 was determined to form the portal cap (18, 19), sealing DNA-filled capsids, and a similar role for pUL77 at the HCMV portal vertex was postulated recently (20, 21). Although similar in size, HCMV capsids must accommodate a much larger genome than α-herpesviruses (240 kbp versus ~150 kbp), and thus are under much higher pressure due to the densely packed genome (22, 23). It was therefore proposed that in HCMV, the function of the α-herpesviral CVSC is taken over by the β-herpesvirus-specific, essential tegument protein pp150 which encloses the capsids as a tight net-like layer, contacting both the hexons and the penton vertices (Fig. 1) (23–25). Consistent with this hypothesis, a Δ*pp150* mutant produced A, B, and C capsids, yet exhibited few cytoplasmic nucleocapsids (26). In addition, the incorporation of pp150 into capsids was reported to be dependent on SCP (27). Until recently, it was unknown whether HCMV pUL77 and pUL93 are located on pentons similar to their α-herpesviral counterparts, particularly since penton occupation by pp150 seemed to exclude simultaneous incorporation of pUL77/pUL93. Recently, cryo-electron microscopy (EM) studies have demonstrated that in approximately half of HCMV particles, some pp150 molecules at the penton vertices are replaced by pUL77/pUL93, although occupancy with pUL77/pUL93 is low (20). To add to this complexity, it has also become evident that SCP, which was initially regarded to be located only on hexons, is also present on pentons (23). Hence, penton vertices have to accommodate a variety of viral proteins whose interplay is only partially understood. In this context, it is important to mention that all cryo-EM data are based on extracellular virions that were stripped from the envelope and outer tegument proteins by treatment with detergents, which can lead to the loss of other tegument and capsid-associated proteins. Also, the protein composition of nuclear capsids may differ from that of extracellular virus particles because the complex mechanism underlying nuclear egress may include tegumentation and detegumentation steps as well as rearrangement of capsid-associated proteins (10, 28). High-resolution cryo-EM analysis of nuclear capsids, however, has not yet been achieved, mainly due to the low number of capsids produced in HCMV-infected cells. In addition, protein regions that are intrinsically disordered cannot be resolved by cryo-EM; this, for instance, is the case for the N-terminal portion of SCP or the C-terminal half of pp150.

In this study, we investigated how deletion of the ORFs encoding pUL77, pUL93, pp150, or SCP influences the protein composition of nuclear HCMV capsids. For this, we generated HCMV bacterial artificial chromosomes (BACs) lacking the respective ORFs and employed a previously established transfection technique, adenofection (29), which allows us to directly study the consequences of deletions in essential genes in BAC-transfected cells. By combining this highly efficient transfection approach with subsequent isolation of nuclear capsids, we found that the lack of SCP severely impairs capsid assembly due to drastically diminished MCP levels. To examine whether a similar effect might explain the previously observed dominant negative effect of an SCP-EGFP (enhanced green fluorescent protein) fusion protein on virion production (3), we included the respective HCMV mutant expressing SCP-EGFP in our analyses. Interestingly, SCP-EGFP did not affect MCP levels, but acted on a step subsequent to capsid formation. Our results elucidate, for the first time, the biological role of the SCP-MCP interaction in the context of virus infection and clarify why SCP is essential in HCMV. Finally, our findings point to new drug-targetable steps in the HCMV assembly process.

## RESULTS

### Protein composition of nuclear HCMV capsids is unaltered in the absence of the capsid-associated proteins pp150, pUL77, and UL93.

**FIG 2:**
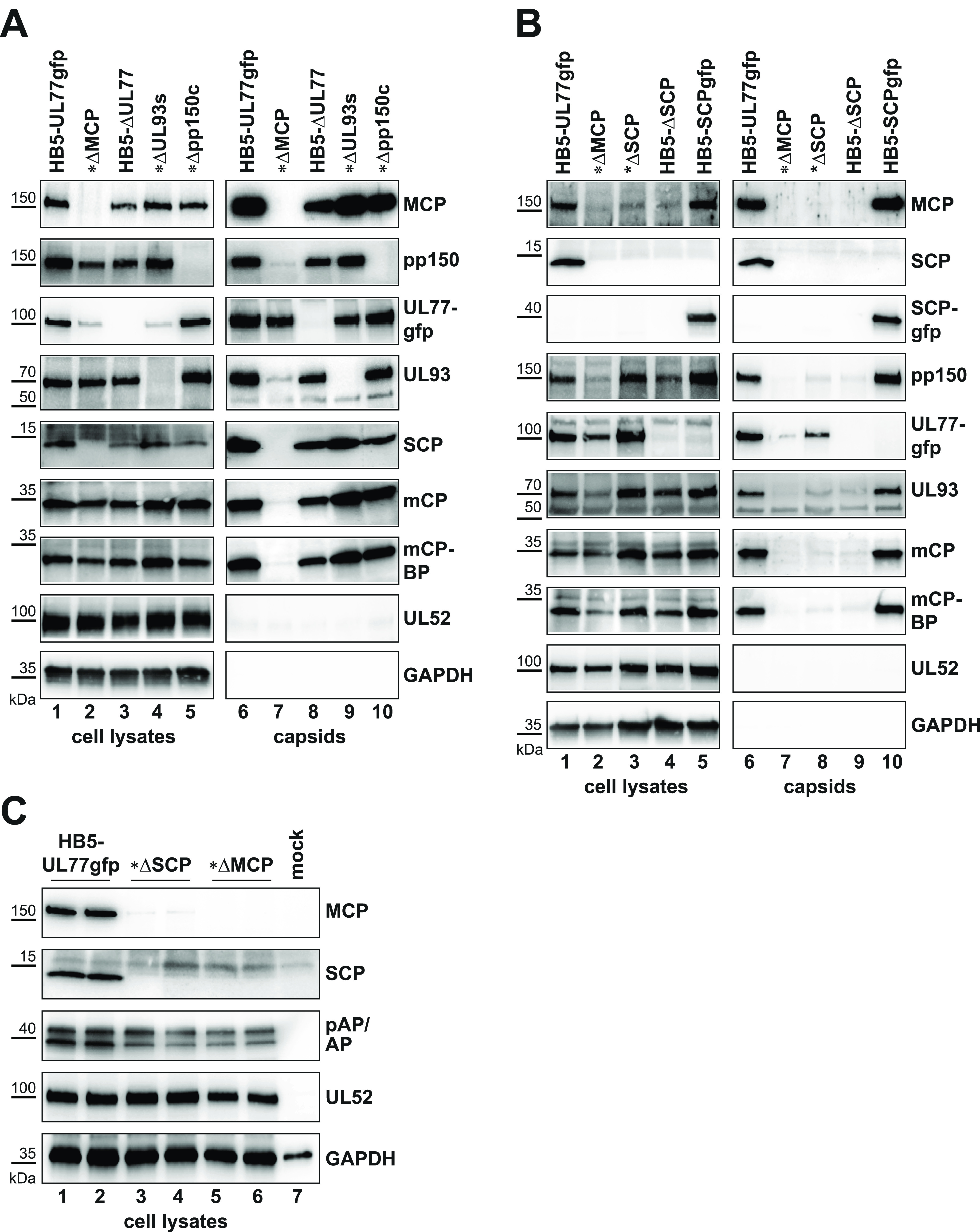
Analysis of nuclear capsids obtained from different HCMV bacterial artificial chromosome (BAC) mutants. (A and B) RPE-1 cells were adenofected with the HCMV BAC HB5-UL77gfp or the indicated BAC mutants, either lacking the denoted ORFs (encoding MCP, pUL77, pUL93, pp150, or SCP) or expressing an SCPgfp fusion protein. Five days later, capsids were purified from the nuclei of adenofected cells and analyzed by immunoblotting using the antibodies listed on the right (GFP antibody for UL77gfp and SCPgfp). Whole-cell lysates prior to capsid isolation are also shown. Here, 1% of the cell lysates and 10% of the capsid samples were loaded. For each protein, identical exposure times were applied for cell lysates and capsids (except for SCP, where the signals obtained with capsids were too strong compared to the cell lysates). Mutants based on the HB5-UL77gfp BAC backbone are marked by asterisks, and HB5 denotes the original BAC-cloned HCMV genome. Note that the pUL93 antibody exhibits some cross-reactivity with a ~50-kDa cellular protein, as described previously (12). (C) Mutual dependence of SCP and MCP on the levels of the respective other protein, and effects on pAP/AP. RPE-1 cells were adenofected with the indicated HCMV BACs, and 4 days later whole-cell lysates were examined by immunoblotting using the specified antibodies. Detection of pUL52 served as a control for comparable adenofection efficiencies.

HCMV procapsids are assembled in the cell nucleus and further develop into either DNA-filled C capsids or empty A and B capsids (Fig. 1). To investigate whether the absence of either pp150, pUL77, or pUL93 has an effect on capsid association of the respective other proteins, we transfected RPE-1 cells (which are fully permissive for HCMV infection) with HCMV BAC genomes lacking the corresponding ORFs by employing the adenofection technique described earlier (29). The HCMV BAC mutants used in this study are listed in Table S1 in the supplemental material. Five days posttransfection, nuclear capsids were isolated from the adenofected cells using an established protocol (12) and analyzed by immunoblotting, along with aliquots of whole-cell lysates taken before capsid preparation (Fig. 2A). Successful isolation of capsids is evident from the detection of all capsid proteins of interest in cells adenofected with the HB5-UL77gfp BAC, which served as positive control (Fig. 2A, lane 6). The absence of pUL52 and GAPDH (glyceraldehyde-3-phosphate dehydrogenase) indicated the purity of the HB5-UL77gfp capsid sample and all other capsid preparations (Fig. 2A, lanes 6 to 10), as reported previously (12). pUL52 is a nuclear viral protein required for HCMV genome packaging (30), yet not a major structural component of capsids or virions (12, 31). As negative control, we used the HB5-UL77gfp-ΔMCP BAC, in which the ORF encoding the major capsid protein is disrupted (12). Hence, no capsids are produced upon transfection, and protein bands observed in the capsid preparation of HB5-UL77gfp-ΔMCP-adenofected cells are considered background. As shown in Fig. 2A (lane 7), pUL77gfp displayed a strong signal in the absence of MCP, and therefore no conclusion could be drawn about the association of pUL77 with capsids. The results obtained with the HB5-ΔUL77 BAC showed that pUL77 is not necessary for capsid binding of pp150, pUL93, or SCP (Fig. 2A, lane 8). Likewise, capsids lacking pUL93 contained ample amounts of pp150 and SCP (Fig. 2A, lane 9). These data corroborate previous reports indicating that pp150 is present on B capsids (12, 32), which are the only capsid form detectable in the absence of pUL77 or pUL93 (11, 12). On the pp150-null capsids, both pUL93 and SCP were detected, indicating that pp150 is not necessary for pUL93 or SCP capsid attachment (Fig. 2A, lane 10). In conclusion, deletion of either UL77, UL93, or UL32 (encoding pp150) did not have obvious effects on the protein composition of nuclear HCMV capsids.

10.1128/mbio.01007-22.5 TABLE S1 HCMV BACs used in this study. Individual ORFs were either completely or partially deleted (depending on their vicinity to or overlap with neighboring ORFs) in some mutants concomitantly with the introduction of stop codons. HB5-ΔUL77 was constructed analogously to the previously published HG-ΔUL77 (12), except that the HB5 BAC does not express EGFP. HB5-UL77gfp-ΔUL93s differs from HB5-UL77-mGFP-3-ΔUL93 (expressing a C-terminally truncated pUL93 version [12]) in that two stop codons were inserted after the alanine codon at position 16 of the UL93 ORF, thereby completely abrogating UL93 expression (data not shown). Download Table S1, DOCX file, 0.03 MB. Copyright © 2022 Borst et al. 2022 Borst et al. https://creativecommons.org/licenses/by/4.0/ This content is distributed under the terms of the Creative Commons Attribution 4.0 International license.

### An SCP-EGFP fusion protein is compatible with capsid assembly, yet absence of SCP impairs capsid formation.

We have shown previously that an SCP-EGFP fusion protein exerts a dominant negative effect on the production of infectious progeny (3). To examine whether this is due to interference with HCMV capsid assembly, we adenofected RPE-1 cells with the HB5-SCPgfp BAC, followed by preparation of nuclear capsids. As seen in Fig. 2B (lane 10), capsids were obtained from the transfected cells, and the SCP-EGFP fusion protein, as well as pUL93, was associated with these capsids. Since it has been reported that pp150 attachment to capsids is impaired after ribozyme-mediated knockdown of SCP expression (27), we examined whether the SCP-EGFP protein hinders pp150 incorporation into capsids. However, this was not the case, as pp150 was abundantly detected on HB5-SCPgfp BAC-derived capsids (Fig. 2B, lane 10). Next, we asked whether pp150 can bind to capsids in the complete absence of SCP, as the reported ribozyme inhibition did not entirely abolish SCP expression (27). To this end, RPE-1 cells were adenofected with either HB5-ΔSCP or HB5-UL77gfp-ΔSCP and the transfected cells were subjected to the capsid purification protocol. Surprisingly, the signals of core capsid proteins were weak and barely different from the background (Fig. 2B, compare lane 7 to lanes 8 and 9), indicating that almost no capsids were obtained with the SCP-null mutants. Notably, when we compared the whole-cell lysates of the adenofected cells (before capsid preparation), it appeared that the lack of SCP resulted in strongly decreased MCP levels (Fig. 2B, lanes 3 and 4), and, vice versa, that deletion of MCP led to a complete loss of SCP (Fig. 2B, lane 2). In contrast, other viral proteins such as pUL52, the triplex proteins (mCP/mCP-BP), and pUL93 remained largely unaffected (Fig. 2B, compare lanes 3 and 4 to lane 1). To verify these findings, we performed transfections in duplicate using the SCP- and MCP-deletion BACs and the parental BAC HB5-UL77gfp, followed by immunoblotting utilizing 10-fold greater amounts of cell lysate than those shown in Fig. 2B. Indeed, in the absence of SCP, MCP levels were drastically reduced (Fig. 2C, lanes 3 and 4), and when MCP was lacking SCP became undetectable (Fig. 2C, lanes 5 and 6; pUL52 expression was used to control for comparable adenofection efficiencies). These data hint at mutual roles of SCP and MCP for establishing appropriate protein levels of the other.

Another major component required for capsid formation is the assembly protein precursor pAP. pAP and the related protease precursor pPR form a complex with MCP and SCP prior to transfer into the cell nucleus (Fig. 1) (7, 8). We therefore examined whether pAP is affected by the absence of SCP or MCP. Two bands were detected by the pAP-specific antibody, with the lower band belonging to the assembly protein (AP), which is generated upon cleavage of pAP by the self-activated protease. Smaller amounts of pAP and AP were detected in RPE-1 cells adenofected with the SCP- and MCP-null BACs compared to those in cells which had received the parental BAC (Fig. 2C, panel 3); however, the effect was less pronounced than that observed for MCP or SCP. Notably, in the samples obtained from HB5-UL77gfp-transfected cells, the band representing AP was stronger than that of pAP (Fig. 2C, lanes 1 and 2). This is consistent with cleavage of pAP, which is known to induce its dissociation from MCP, thereby promoting capsid angularization (7, 8). In contrast, this was not seen in cells transfected with the SCP- and MCP-null BACs, where either the upper pAP band was the stronger one or the pAP/AP signals were of comparable intensity (Fig. 2C, lanes 3 to 6). In conclusion, these findings are in line with the assumption that capsid formation is strongly reduced in the absence of SCP due to severely diminished MCP levels.

### Absence of SCP primarily influences MCP levels.

**FIG 3:**
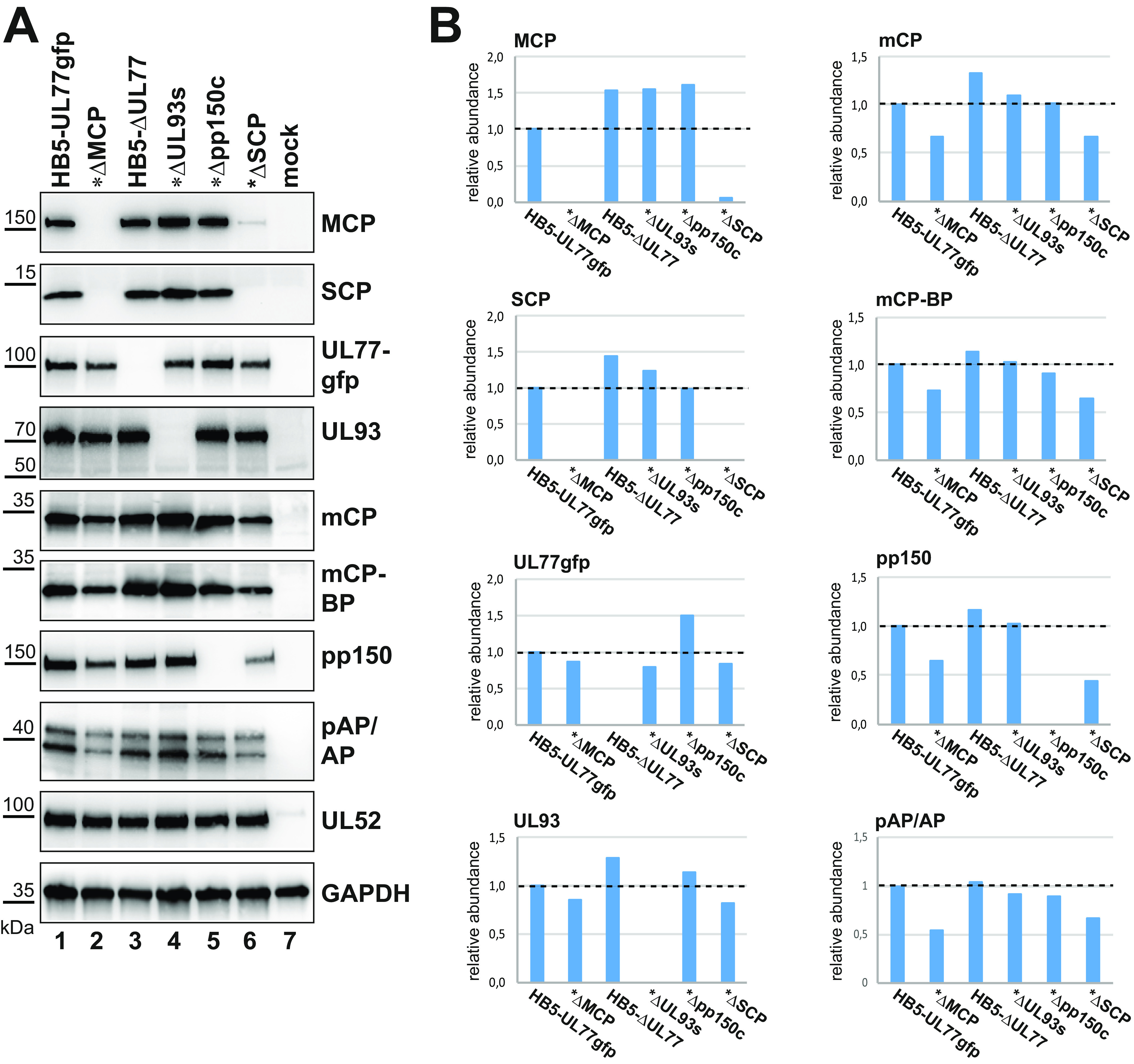
Comparison of capsid protein levels of HCMV BAC mutants carrying deletions in different essential genes. RPE-1 cells were adenofected with the HCMV BACs indicated (asterisks denote the HB5-UL77gfp BAC as the backbone used for the corresponding deletion BACs) and incubated for an additional 4 days. The ΔUL93s BAC harbors two stop codons after amino acid A16, and in the Δpp150c BAC mutant the whole pp150 ORF is deleted (as compared to the previously published ΔUL93 and Δpp150 BACs [12]; see Materials and Methods and Table S1). (A) Whole-cell lysates were prepared and subjected to immunoblotting with the specified antibodies (GFP antibody for UL77gfp). (B) Immunoblotting signals were quantified using Image Lab 6.0.1 software (Bio-Rad) and normalized to the pUL52 signals (to adjust for comparable transfection efficiencies). *y* axis shows relative amounts of the respective viral protein indicated above each panel; *x* axis shows different mutant BACs transfected as shown in panel A.

Next, we analyzed all BAC mutants in parallel to thoroughly examine the levels of the various capsid and capsid-associated proteins when SCP is missing; furthermore, we checked whether similar effects occur in the absence of other capsid components. Lysates of cells adenofected with the deletion BACs or the parental HB5-UL77gfp BAC were examined by immunoblotting, and the band intensities of the different proteins were quantified (Fig. 3). As observed previously, MCP was substantially diminished when SCP was missing (Fig. 3A, lane 6), and SCP was basically absent in ΔMCP-transfected cells (Fig. 3A, lane 2). pUL77gfp and pUL93 were marginally influenced in ΔSCP- and ΔMCP-adenofected cells. The triplex proteins mCP and mCP-BP were slightly reduced (compare lane 1 to lanes 2 and 6, and Fig. 3B), while pp150 was present at somewhat lower levels in the SCP-null mutant. Notably, neither MCP nor SCP levels were decreased upon deletion of UL77, UL93, or pp150 (Fig. 3A, lanes 3 to 5). We conclude from these data that the mutual dependence of SCP and MCP for establishing the respective other protein levels is largely specific and does not involve other capsid proteins. Regarding the scaffolding proteins, pAP/AP amounts in ΔUL77-, ΔUL93-, and Δpp150-adenofected cells were comparable to those detected with the parental BAC (Fig. 3A, lanes 3 to 5, and Fig. 3B), and a bit lower in the absence of MCP or SCP (Fig. 3A, lanes 2 and 6; see also Fig. 2C). Importantly, higher AP signals indicating pAP cleavage were only observed for BAC mutants known to complete capsid assembly (UL77-, UL93-, and pp150-null mutants, lanes 3 to 5 [11, 12, 26]), while this was not seen when MCP or SCP was missing (Fig. 3A, lanes 2 and 6), supporting the findings described above.

### Lack of SCP or presence of SCP-EGFP affects nuclear capsid types.

**FIG 4:**
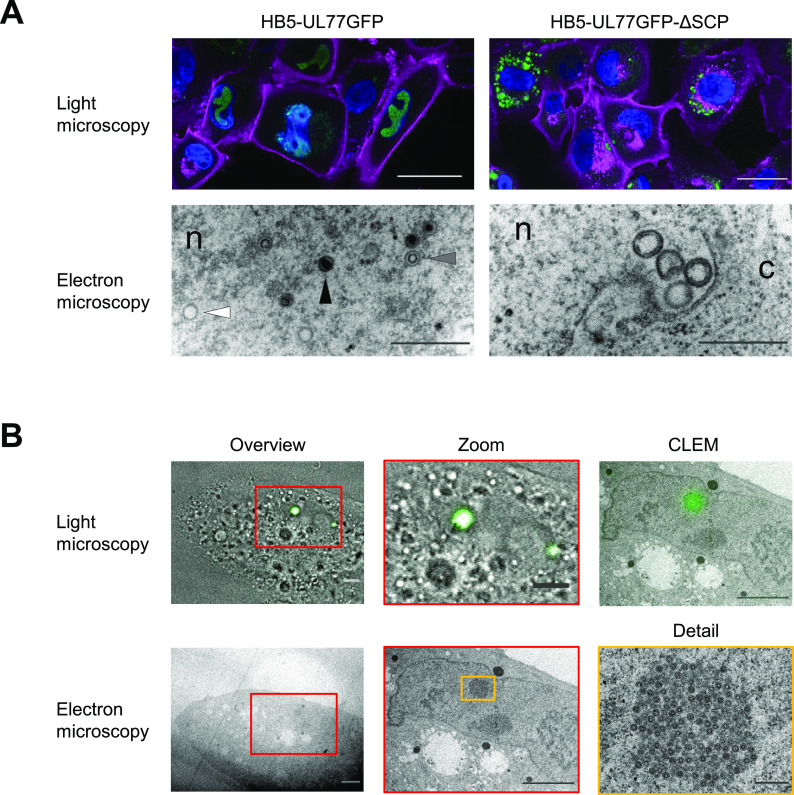
Correlative light and electron microscopy (CLEM) analysis of RPE-1 cells adenofected with the parental HCMV-UL77gfp BAC or the SCP-deletion BAC derived thereof (A), or with the HB5 BAC expressing an SCPgfp fusion protein (B). (A) Five days after transfection, cells were fixed, subjected to Hoechst and wheat germ agglutinin staining, and further processed for CLEM as described in Materials and Methods. White arrowhead, A capsid; gray arrowhead, B capsid; black arrowhead, C capsid. n, nucleus; c, cytoplasm. Scale bars = 50 μm (light microscopy) and 500 nm (electron microscopy). (B) SCPgfp BAC-adenofected cells were analyzed by light and electron microscopy on day 5 posttransfection. Scale bars = 5 μm (overviews, zooms, CLEM) and 500 nm (detail).

Since capsid purification by gradient centrifugation pointed to strongly diminished capsid yields in the absence of SCP (c.f. Fig. 2B), we performed correlative light and electron microscopy (CLEM) studies to examine whether any capsids could be detected directly in cells adenofected with the HB5-UL77gfp-ΔSCP BAC. Transfected cells were identified via fluorescence of pUL77gfp. While pUL77gfp was found to be nuclear in cells adenofected with the parental BAC, as observed previously (12), in cells adenofected with the SCP deletion BAC the pUL77gfp fusion protein was apparently mislocalized to cytoplasmic dots (Fig. 4A). Cells scoring positive for pUL77gfp expression were selected for electron microscopy, and all capsid types (A, B, and C) were found in the nuclei of HB5-UL77gfp-adenofected cells (Fig. 4A, left; 15% A, 52% B, and 34% C capsids of 104 total capsids, data not shown). Notably, when SCP was lacking only, very few, spherical structures were detected in the nuclei, potentially representing aberrant procapsids or scaffold assemblies (Fig. 4A, right), whereas no typical A, B, or C capsids were seen. Thus, the absence of SCP led to a defect in capsid formation, most likely due to its pronounced effect on MCP levels. This finding also explains the cytoplasmic distribution of pUL77gfp, which is typically associated with nuclear A, B, and C capsids (12).

In contrast to the ΔSCP BAC, capsids could be readily purified from HB5-SCPgfp-adenofected RPE-1 (c.f. Fig. 2B), although it is known that no infectious progeny can be recovered (3). To examine which capsid forms are generated in these cells, transfected RPE-1 cells were identified via the green fluorescent SCPgfp dots in the nucleus (3) and analyzed by CLEM. Remarkably, these nuclear dots turned out to contain a plethora of densely packed B capsids arranged in membrane-less, paracrystalline structures (Fig. 4B). However, no A or DNA-filled C capsids were seen (2,738 total capsids, all found in clusters as described in Fig. 4B), demonstrating that no genome packaging had occurred, and thereby explaining—together with the abnormal intranuclear accumulation of SCPgfp capsids—how SCPgfp disrupts the viral infection cycle.

### SCP and MCP levels can be rescued by *trans*-complementation.

**FIG 5:**
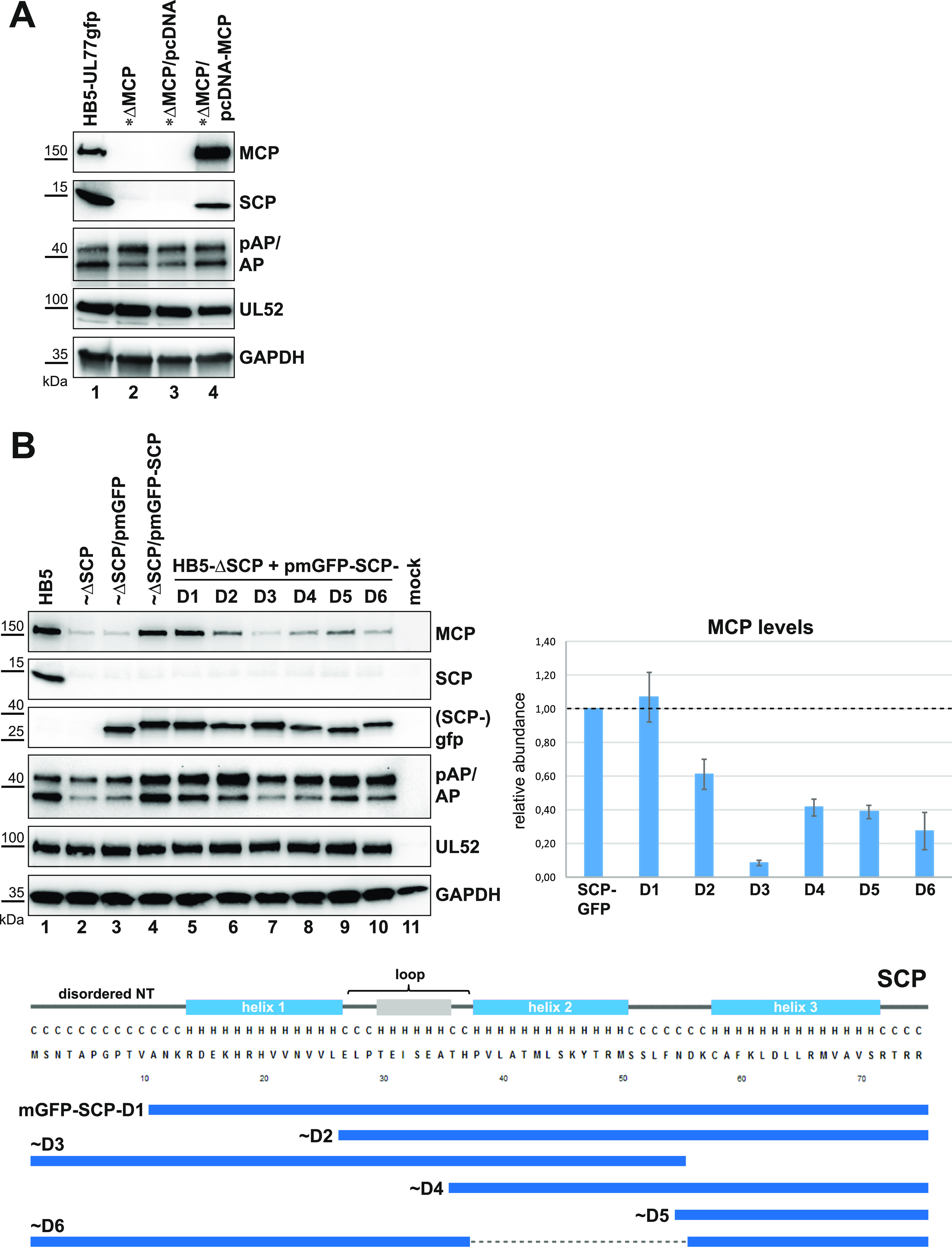
*Trans*-complementation of the MCP and SCP deletion BACs and determination of SCP domains which influence MCP levels. (A) Restoration of SCP expression after co-adenofection of the MCP-null BAC and an MCP-expressing plasmid (pcDNA-MCP). RPE-1 cells were adenofected with the parental HB5-UL77gfp BAC or the corresponding MCP deletion BAC, either alone or in combination with the MCP-expressing plasmid or the empty vector (pcDNA). Cell lysates were prepared 4 days later and analyzed by immunoblotting using the indicated antibodies. (B) Evaluation of MCP expression following co-adenofection of the HB5-ΔSCP BAC with an mGFP-expressing plasmid (pmGFP), an mGFP-SCP-expressing plasmid (pmGFP-SCP), or truncation mutants of pmGFP-SCP (D1 to D6). Left side: on day 4 after transfection, cell lysates were examined by immunoblotting using the antibodies shown (GFP for SCPgfp). Right side: MCP signals from three independent experiments were quantified (first normalized to the corresponding pUL52 signals, then compared to the mGFP-SCP signals obtained upon co-transfection of the indicated plasmids). Bottom half: scheme depicts the truncated mGFP-SCP variants, with the secondary structure prediction (http://bioinf.cs.ucl.ac.uk/psipred/) and the SCP amino acid sequence given above. NT, N terminus; loop, putative helix recently identified to represent a loop structure (see the text for details); C, coil; H, helix.

We reasoned that if SCP and MCP levels are dependent on the presence of the respective other protein, providing the missing protein in *trans* should rescue the phenotype of the corresponding BAC mutant. To address this question, we performed co-adenofection of the MCP deletion BAC with an MCP-expressing plasmid (pcDNA-MCP) and checked for SCP expression in the co-transfected cells. High MCP expression was obtained with pcDNA-MCP which, moreover, was able to restore expression of SCP, albeit not to the same level as that achieved with the parental BAC HB5-UL77gfp (Fig. 5A, compare lane 1 to lane 4). In contrast, co-transfecting the empty pcDNA vector did not have an effect on SCP (Fig. 5A, lane 3). This result further substantiates our discovery that SCP levels are dependent on MCP. Notably, MCP provided by plasmid expression also resulted in increased AP levels (due to pAP cleavage) compared to those in cells adenofected with the MCP-null BAC alone or co-adenofected with the empty pcDNA plasmid (Fig. 5A, lanes 2 to 4).

In a similar approach, we generated plasmid pmGFP-SCP expressing SCP fused in-frame to the C terminus of monomeric EGFP, and co-adenofected RPE-1 cells with this plasmid and the HB5-ΔSCP BAC. Indeed, MCP expression was rescued efficiently by the mGFP-SCP fusion protein (Fig. 5B, lane 4), while this was not the case when the parental pmGFP plasmid was used (Fig. 5B, lane 3). Besides this, conversion of pAP into AP was markedly enhanced in the presence of mGFP-SCP, whereas this was not observed with HB5-ΔSCP alone or upon co-transfection of the pmGFP plasmid (Fig. 5B, compare lanes 2 to 4). In conclusion, these data strongly support our hypothesis that SCP is crucial to retaining appropriate MCP levels.

### SCP domains required to preserve MCP levels.

Next, we wanted to narrow down the regions of SCP responsible for the effect on MCP levels. HCMV SCP is predicted to consist of three helices and an unstructured N-terminal portion (Fig. 5B, lower part), which was recently confirmed by cryo-EM (23). Based on plasmid pmGFP-SCP, we generated three constructs in which different parts of the SCP N-terminal half were truncated. Plasmid pmGFP-SCP-D1 lacks the intrinsically disordered N terminus (NT), pmGFP-SCP-D2 additionally lacks helix 1, and in pmGFP-SCP-D4 the loop region is absent as well (Fig. 5B, lower half). To investigate the role of helix 3, this region was either deleted (pmGFP-SCP-D3) or expressed on its own (pmGFP-SCP-D5). Similarly, to analyze the importance of helix 2, plasmid pmGFP-SCP-D6 carrying a deletion of the respective sequence was constructed. RPE-1 cells were then co-adenofected with the HB5-ΔSCP BAC and the different expression plasmids. Effects on MCP levels were examined by immunoblotting (Fig. 5B, upper left) and MCP signals from three independent experiments were quantified (Fig. 5B, bar graph). Co-transfection of pmGFP-SCP-D1 could rescue MCP levels to the same extent as the full-length pmGFP-SCP plasmid (Fig. 5B, upper left [lanes 4 and 5]), indicating that the disordered SCP NT is not required for regulation of MCP abundance. In contrast, pmGFP-SCP-D3 did not restore MCP levels, pointing to an important role of helix 3, which is in accordance with helix 3 mediating SCP interaction with MCP (23, 33). However, providing SCP helix 3 alone could not fully restore MCP levels (pmGFP-SCP-D5). Constructs pmGFP-SCP-D2, -D4, and -D6 had intermediate effects on MCP levels (Fig. 5B, lanes 6, 8, and 10, and bar graph). Taken together, these data imply that SCP helix 3 is essential for preserving stable MCP levels, while helix 1, helix 2, and the loop region contribute to full activity. In line with this, efficient pAP cleavage was seen in the presence of full-length mGFP-SCP, and to varying degrees for the truncated versions.

**FIG 6:**
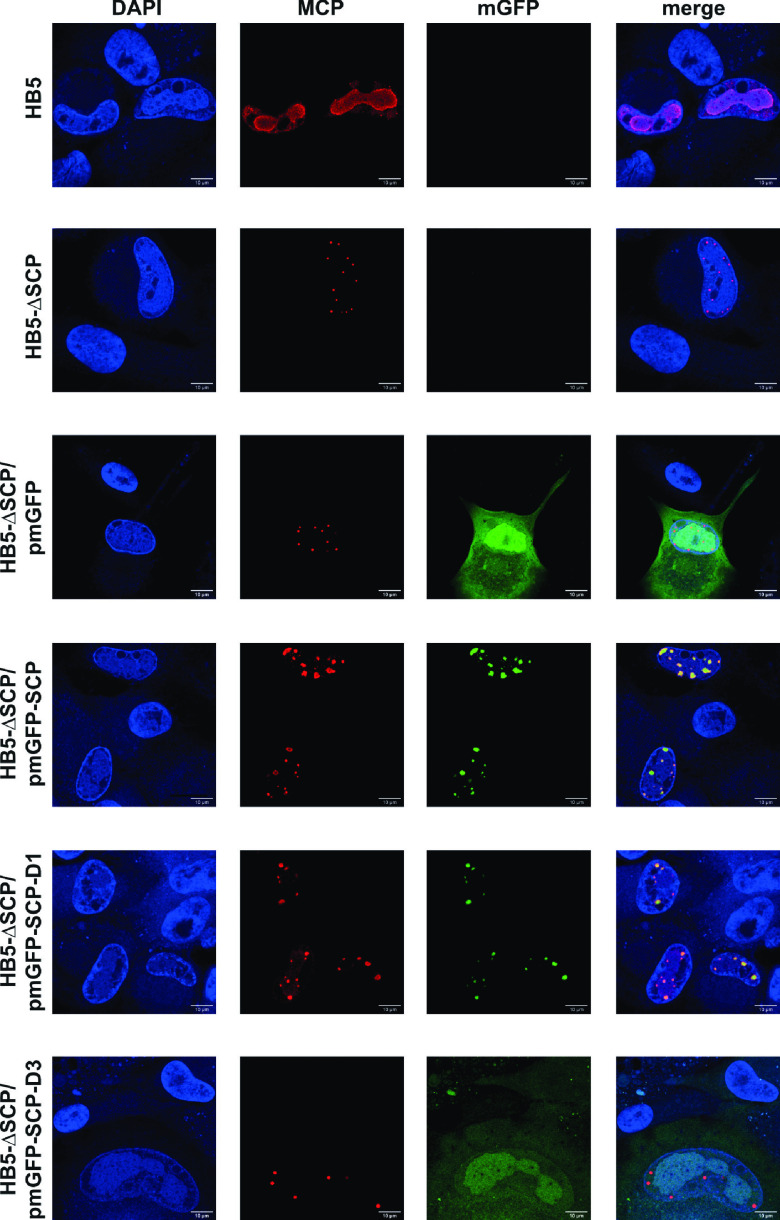
MCP localization in the absence of SCP or after complementation with either mGFP, the mGFP-SCP fusion protein, or the truncated versions mGFP-SCP-D1 and -D3. Adenofected RPE-1 cells were fixed on day 4 posttransfection, followed by labeling with an MCP antibody (as described previously [12]) and DAPI (4′,6-diamidino-2-phenylindole). Confocal laser scanning microscopy was performed using a Leica inverted-3 microscope, and images were further processed using Fiji Image J software. Scale bar = 10 μm.

Finally, we used immunofluorescence microscopy to check for the presence and intracellular localization of MCP in the absence of SCP, or upon coexpression of the different mGFP-SCP variants (Fig. 6 and Fig. S1 in the supplemental material). While in HB5-adenofected RPE-1 cells MCP was detected throughout the nuclear viral replication compartments, as expected (Fig. 6, lane 1), in HB5-ΔSCP-transfected cells MCP was found only in tiny nuclear dots (Fig. 6, lane 2). The same was seen upon co-adenofection of HB5-ΔSCP and the mGFP-expressing plasmid (Fig. 6, lane 3). When the plasmid expressing the full-length mGFP-SCP fusion protein was co-adenofected, the MCP signals were larger and patchy in shape. Distinct co-localization with the mGFP-SCP fusion protein was observed (Fig. 6, lane 4), and a very similar pattern was seen with pmGFP-SCP-D1 expressing the SCP variant without the disordered N terminus (see Fig. 5B). Likewise, the mGFP-SCP truncated versions D2, D4, D5, and D6, displaying an intermediate phenotype concerning their influence on MCP levels (Fig. 5), also co-localized with MCP in small nuclear patches (Fig. S1). In contrast, co-adenofection of HB5-ΔSCP with pmGFP-SCP-D3, which could not rescue MCP amounts (Fig. 5B), resulted in the mGFP-SCP-D3 variant being diffusely distributed throughout the cell, with MCP again being present in small nuclear punctae (Fig. 6, lane 6). Quantification of MCP signals in individual cell nuclei revealed that complementation of the ΔSCP mutant with either pmGFP-SCP or pmGFP-SCP-D1 yielded comparable MCP levels which were, moreover, similar to those of cells adenofected with the parental HB5 BAC (Fig. S2). Conversely, RPE-1 transfected with HB5-ΔSCP alone or in combination with pmGFP or pmGFP-SCP-D3 displayed considerably lower MCP signals. These results are in agreement with the immunoblotting data (Fig. 5B) and corroborate our finding that the absence of SCP has a strong impact on MCP levels, with SCP helix 3 being of major importance.

10.1128/mbio.01007-22.1 FIG S1 Intracellular localization of major capsid protein (MCP) upon co-adenofection of the ΔSCP BAC (bacterial artificial chromosome) with expression constructs pmGFP-SCP-D2, -D4, -D5, and -D6. Cells were labelled with an MCP antibody and DAPI (4′,6-diamidino-2-phenylindole) on day 4 post-adenofection and analyzed by confocal laser scanning microscopy as shown in Fig. 6. Scale bar = 10 μm. Download FIG S1, PDF file, 0.2 MB. Copyright © 2022 Borst et al. 2022 Borst et al. https://creativecommons.org/licenses/by/4.0/ This content is distributed under the terms of the Creative Commons Attribution 4.0 International license.

10.1128/mbio.01007-22.2 FIG S2 Quantification of MCP immunofluorescence signals of adenofected RPE-1 cells from the experiment shown in Fig. 6. Fiji Image J software was used to measure MCP signals in randomly chosen cell nuclei (identified by DAPI staining). An arbitrary cutoff (12 arbitrary units, AU) was used to exclude signals from non-transfected cells. Black bars indicate mean values. Download FIG S2, EPS file, 1.8 MB. Copyright © 2022 Borst et al. 2022 Borst et al. https://creativecommons.org/licenses/by/4.0/ This content is distributed under the terms of the Creative Commons Attribution 4.0 International license.

## DISCUSSION

The main finding of this study is a novel, unexpected function of the HCMV small capsid protein in regulating the abundance of the major capsid protein. We began by analyzing how the absence of one of the capsid-associated proteins pUL77, pUL93, or pp150 affects binding of the respective other proteins to capsids. These proteins are expected to be arranged around pentons, and in the case of pp150, also around hexons, connecting the capsid floor with the tips of the capsomers, most likely in order to stabilize HCMV capsids. Since SCP is located on the tips of capsomers, this protein was included in analyses as well. In fact, recent cryo-EM data have implied that SCP contacts pp150 molecules (23, 27).

The rapid comparison of HCMV genomes each lacking one of the mentioned genes became possible only through application of our high-efficiency BAC-adenofection approach (29). As described previously, this technique is particularly useful for analyzing late stages of the infection cycle, such as capsid assembly. It works without the need to create complementing cell lines for each mutant, which can be cumbersome because the missing viral protein has to be expressed with appropriate kinetics and quantity during the prolonged HCMV infection cycle. Moreover, adenofection proved efficient enough to isolate nuclear capsids from a reasonable number of cells.

Our experiments indicate that there are no major changes in capsid composition when either pUL77, pUL93, or pp150 is lacking. However, the immunoblot analyses do not permit concluding about the configuration of the respective proteins on capsids. Further insight into this aspect must await technical advances in imaging techniques, particularly since the low number of nuclear HCMV capsids hampers examination by high-end cryo-EM. In view of the recent observation that in about 50% of capsids (obtained from extracellular virions) some of the pp150 molecules are replaced by the CVSC components pUL77/pUL93 (20), one might have expected that pp150-null capsids contain higher levels of pUL77 and pUL93. We did not observe this for pUL93 and could not draw a conclusion for pUL77 because of high signals that occurred even when capsid assembly was abolished by deletion of MCP. This hints at pUL77 multimers of high density that could pass through the sucrose cushion during capsid purification. In fact, multimerization of pUL77 has been proposed (21); meanwhile, it has become clear that pUL77 oligomers are sealing the HCMV portal (20). Presumably, the generation of even larger pUL77 structures is favored in the absence of capsids.

Steric hindrance of pp150 capsid association by the SCP-EGFP fusion protein might also have been expected in cells transfected with the HCMV-SCPgfp BAC. However, the immunoblot analysis showed that pp150 levels were not affected. CLEM revealed that in these cells, capsids were assembled, but we exclusively saw empty B capsids accumulating in paracrystalline clusters. This finding sheds light on why the HCMV-SCPgfp BAC genome does not give rise to infectious progeny. The green fluorescent dots resulting from the agglomerated nuclear B capsids are reminiscent of HSV-1 mutants expressing fluorescently tagged VP26 proteins (the ortholog of HCMV SCP). However, in case of the HSV-1 mutants, these dots originate from protein aggregates and not from capsids (34). Although EGFP tends to dimerize, it is unlikely that this property caused the capsid accumulations, because another HCMV mutant expressing SCP fused to monomeric RFP exhibited the same phenotype as HCMV-SCPgfp (Fig. S3). The ability of such SCP fusion proteins to act in a dominant negative manner, i.e., blocking virus growth even when native SCP is provided simultaneously (3), may be exploited for pharmacological intervention.

10.1128/mbio.01007-22.3 FIG S3 Correlative light and electron microscopy (CLEM) of RPE-1 cells adenofected with a human cytomegalovirus (HCMV) mutant expressing a small capsid protein (SCP)-mRFP fusion protein. mRFP was inserted between SCP amino acids 4 and 5 (T/A). Transfected cells were fixed on day 4 post-adenofection and processed for CLEM as described in Materials and Methods. Download FIG S3, PDF file, 0.4 MB. Copyright © 2022 Borst et al. 2022 Borst et al. https://creativecommons.org/licenses/by/4.0/ This content is distributed under the terms of the Creative Commons Attribution 4.0 International license.

Contrary to the effect of the SCP-EGFP protein, capsid assembly was severely impaired in the absence of SCP. We discovered that this is due to strongly reduced MCP levels when SCP is lacking. The reverse was true as well, as SCP became undetectable upon MCP deletion. Thus, there is a mutual dependence of SCP and MCP for ensuring appropriate protein levels. This effect of SCP on MCP does not occur on a transcriptional level, as reverse transcriptase quantitative PCR experiments showed that MCP RNA levels are not disturbed in the absence of SCP (Fig. S4). Notably, a recent report described that the autophagy receptor SQSTM1 colocalizes with MCP and capsids in the nuclei of infected cells and, furthermore, co-immunoprecipitates with both MCP and SCP as well as other capsid components (35), suggesting their targeting to autophagosomal degradation. These findings point to a posttranslational mechanism regulating SCP and MCP stability.

10.1128/mbio.01007-22.4 FIG S4 Analysis of MCP transcript levels in the ΔSCP and ΔMCP BAC mutants by reverse transcriptase quantitative PCR. Adenofection of RPE-1 and RNA isolation was done as described previously (44) in three biological replicates. One μg of RNA was reverse transcribed using the RevertAid H Minus Reverse Transcriptase kit with oligo-dT priming and including the RNase inhibitor RiboLock according to the manufacturer’s instructions (Thermo Fisher Scientific, Waltham, MA). qPCR reactions were performed using a Rotorgene 6000 qPCR device (Qiagen, Hilden, Germany). Each PCR reaction consisted of SensiMix SYBR Hi-ROX kit (Bioline), 50 ng cDNA, and forward and reverse primers (10 μM) in a final volume of 10 μL. Amplification conditions were as follows: denaturation and polymerase activation at 95°C for 7 min, followed by 40 cycles, with 1 cycle consisting of 10 s at 95°C, 40 s at 57°C, and 10 s at 72°C (three technical replicates). MCP RNA amounts were normalized to those of UL52 transcripts as described elsewhere (44). Means ± standard error of the mean are shown. Data were analyzed by unpaired *t* test using the Rotor gene assay manager software (Qiagen) (ns, not significant). Note that in the ΔMCP mutant, the MCP open reading frame (ORF) was disrupted by insertion of two stop codons, leaving the primer binding sites unaffected. Download FIG S4, EPS file, 0.9 MB. Copyright © 2022 Borst et al. 2022 Borst et al. https://creativecommons.org/licenses/by/4.0/ This content is distributed under the terms of the Creative Commons Attribution 4.0 International license.

Previously, the interaction between SCP and MCP has been studied in transiently transfected cells or in mammalian-two-hybrid experiments (M2H), whereas we have now analyzed the role of this interaction in the context of virus infection. The M2H experiments disclosed a crucial role of the SCP C terminus for MCP interaction (33), which was meanwhile verified by structural data identifying that SCP helix 3 fits snugly into an SCP-binding cleft on the tip of the MCP molecule (23). Our discovery that the effect of SCP on MCP levels is dependent on the C-terminal helix corresponds perfectly well to these previous data.

It has been described that in transiently transfected cells, MCP is cytoplasmic and SCP is distributed throughout the cell, and when coexpressed SCP and MCP are retained together in the cytoplasm (33). In HCMV-infected cells, both SCP and MCP are detected in nuclear replication compartments (12), congruent with the fact that their nuclear localization is dependent on the scaffolding proteins (7, 8, 33). We have now found that in the absence of SCP, the nuclear MCP signal is strongly diminished, which is consistent with immunoblotting data of cells transfected with the ΔSCP mutant. However, the residual MCP was not distributed throughout the replication compartments but was located in nuclear punctae. In conclusion, this suggests that MCP undergoes substantial turnover when SCP is absent, yet the remaining MCP is still translocated to the nucleus.

While lack of SCP led to drastically diminished MCP levels, the abundance of the scaffold protein pAP was only moderately affected, yet lower levels of its cleavage product AP were detected. Scaffold cleavage by the self-activated protease is indicative of capsid angularization and occurs simultaneously with genome packaging; however, the mechanism underlying protease activation remains largely unclear (7, 8). Given that low-level pAP cleavage also occurred with the ΔMCP mutant, it appears that some degree of spontaneous protease activity can take place even upon abolishing capsid formation. The ΔSCP mutant also displayed little pAP cleavage, in line with the drastic reduction of capsid formation and the occasional detection of only few spherical structures in the nuclei of ΔSCP-transfected cells, which may correspond to aberrant procapsids or scaffold assemblies. However, upon transient complementation of ΔSCP with the full-length SCP-mGFP fusion protein, pAP cleavage was enhanced, which is in agreement with the simultaneous restoration of MCP levels and our finding that angularized B capsids are produced by the HB5-SCPgfp mutant. Moreover, in cells expressing SCP-mGFP, the nuclear dots displaying an overlap of the GFP and MCP signals were highly similar to the fluorescent patches seen with the HCMV mutant HB5-SCPgfp, thus most likely representing capsid conglomerates.

It has been proposed that HCMV SCP is essential because it mediates incorporation of the innermost tegument protein pp150 into capsids (27). Our data for the SCP-null mutants indicate disruption of capsid assembly at an earlier stage. At first glance, these results seem to be in conflict with each other; however, the experimental settings are different. Dai et al. (27) used ribozyme-mediated inhibition of SCP expression, which was efficient yet not complete. Obviously, then, it makes a difference whether SCP is knocked down or is entirely lacking, as in the ΔSCP mutants examined here. The discovered effect of SCP on MCP levels and capsid assembly appears to be specific for HCMV, because SCP/VP26 is not essential in α-herpesviruses (4), and in Kaposi’s sarcoma-associated herpesvirus (KSHV, a γ-herpesvirus) SCP/ORF65 exerts an important but nonessential role by cementing assembled capsids through bridging of neighboring hexons (5). Hexon bridging was seen neither in HSV-1 nor in HCMV SCP and is virtually impossible for the latter because of its small size (75 aa versus 170 aa in KSHV). In summary, we assigned a novel function to HCMV SCP which further contributes to our understanding of its pivotal role in the viral infection cycle. Finally, tampering with the SCP-MCP interface may lead to the identification of new antiviral inhibitors, which would possess alternative modes of action and broaden the number of drugs available to treat HCMV disease.

## MATERIALS AND METHODS

### BAC mutagenesis.

The HCMV BACs used and generated in this study are all based on the BAC-cloned AD169 genome pHB5 (36) (see Table S1 for an overview of BAC genomes and Table S2 for the primers employed). BAC mutagenesis was achieved by *en passant* mutagenesis (37). HB5-UL77gfp-ΔUL93s was constructed using the primers UL93-KO-stop.for and UL93-KO-stop.rev with HB5-UL77-mGFP-3-ΔUL93 as the backbone (12), and HB5-UL77gfp-Δpp150c with primer pair D-pp150.for and D-pp150.rev and HB5-UL77-mGFP-3 as the backbone (12). HB5-UL77gfp-ΔSCP is also derived from HB5-UL77-mGFP-3 and was generated utilizing the primers SCP-KO.for and SCP-KO.rev. For these three mutant BACs, pori6K-RIT (38) was used as the template for PCR. HB5-SCP-mRFP was obtained from pHB5, with primers SCP-mRFP.for and SCP-mRFP.rev, using pMCMV3-mRFP-in (Messerle, unpublished) as the template. HB5-ΔUL77 was constructed as described elsewhere (39), using pHB5 as the backbone instead of the EGFP-expressing pHG BAC (40).

10.1128/mbio.01007-22.6 TABLE S2 Oligonucleotides used in this study. *P*~: oligonucleotides phosphorylated at the 5′ end. Download Table S2, DOCX file, 0.01 MB. Copyright © 2022 Borst et al. 2022 Borst et al. https://creativecommons.org/licenses/by/4.0/ This content is distributed under the terms of the Creative Commons Attribution 4.0 International license.

### Purification of nuclear HCMV capsids following BAC adenofection.

hTERT-RPE-1 cells (Clontech Laboratories Inc., now TaKaRa Bio, Inc.) were cultivated as described previously (29). One day before adenofection, 3 × 10^6^ cells were seeded into a T175 flask. Adenofection was performed as outlined previously (29). In brief, 7 μg of BAC DNA was diluted in 1.5 mL of HBS buffer (20 mM HEPES, 150 mM NaCl [pH 7.4]), and 50 μL of PEI 2000 solution (0.9 mg/mL in aqua bidestillata [pH 7]) was diluted in 1.5 mL of HBS buffer. The solutions were mixed, and after 20 min at room temperature (RT), 2.7 × 10^10^ particles of a replication-incompetent adenovirus mutant (ΔE4) further inactivated by methoxypsoralen and UV light treatment were added. After incubation of the sample for another 20 min at RT, the transfection complexes were added to the RPE-1 cells sitting in serum-free medium containing 30 μg/mL polymyxin B. Following 5 h of incubation at 37°C/5% CO_2_, cells were washed with phosphate-buffered saline (PBS) and complete growth medium (containing 30 μg/mL polymyxin B) was added. Capsid isolation was done 5 days later using an established protocol (12).

### Small-scale adenofection, co-adenofection, and immunoblotting.

For analysis of whole-cell lysates by immunoblotting, hTERT-RPE-1 cells were seeded into 6-well-plates the day before adenofection (1.7 × 10^5^ cells/well). One μg of BAC DNA was used in combination with 10 μL of PEI 2000 solution and 5.4 × 10^9^ adenovirus particles, and for co-adenofection 1 ng of each expression plasmid was added per sample. Four days post adenofection, cells were scraped into PBS, pelleted by centrifugation at 400 × *g*, and lysed in Roti-Load 1 solution (Carl Roth, Karlsruhe, Germany). Next, 10% of the lysates was analyzed by SDS-PAGE and immunoblotting using the following antibodies: anti-MCP (mouse hybridoma supernatant; kindly provided by Klaus Radsak, formerly University of Marburg, Germany) diluted 1:200, anti-pp150 mouse hybridoma supernatant (this study, see below) diluted 1:100, anti-GFP rabbit monoclonal antibody (MAb) D5.1 XP (Cell Signaling Technology, Danvers, MA) diluted 1:1,000, anti-UL93 mouse hybridoma supernatant (12) diluted 1:100, anti-SCP mouse hybridoma supernatant (kind gift from William Britt, University of Alabama, Birmingham) diluted 1:400, anti-mCP, anti-mCP-BP, and anti-pAP polyclonal rabbit sera (all generously provided by Wade Gibson, Johns Hopkins University, Baltimore, MD) diluted 1:2,000 (anti-mCP, anti-mCP-BP) or 1:5,000 (anti-pAP). The generation of the anti-UL52 MAb (mouse hybridoma supernatant diluted 1:200) was described previously (41), and anti-GAPDH obtained from Cell Signaling Technology (rabbit monoclonal antibody 14C10) was used at 1:2,000. All immunoblots were done using the SuperSignal Western Blot Enhancer kit (Thermo Fisher Scientific, Waltham, MA) according to the manufacturer’s instructions.

### Generation of a pp150-specific antibody.

The DNA sequence encoding the pp150 B cell epitope comprising aa 862 to 1,048 as defined by Vornhagen et al. (42) was PCR-amplified from HCMV BAC pHB5 with the primers UL32-BCE2.for and UL32-BCE2.rev (Table S2) and cloned into pQE-30 (Qiagen, Hilden, Germany) cut with SphI and HindIII using the Gibson Assembly Master Mix following the manufacturer’s recommendations (New England Biolabs [NEB], Ipswich, MA, cat no. E2611S). Expression of the recombinant protein in E. coli, purification, immunization of mice, and generation of hybridoma cultures was performed as described previously (41). Antibodies recognizing the recombinant protein were further evaluated with HCMV-infected and noninfected cells by immunofluorescence microscopy and immunoblotting, and the antibody exhibiting the best signal-to-noise ratio was chosen for subcloning of the corresponding hybridoma culture.

### Correlative light and electron microscopy.

hTERT-RPE-1 cells to be analyzed by CLEM were adenofected in 6-well-plates as described above. Three days post-adenofection, cells were split into 35-mm 500-grid dishes (2 × 10^5^ cells/dish; Ibidi GmbH, Gräfelfing, Germany, cat no. 81166). The next day, cells were washed twice with PBS and fixed by applying 2% paraformaldehyde/2.5% glutaraldehyde in PBS (Science Services GmbH, Munich, Germany, cat no. E15713 and E16220, respectively) for 5 min at RT followed by 55 min at 4°C using the same fixing solution. After two washes with cold PBS, cells were kept at 4°C in 2% paraformaldehyde in PBS overnight. For correlative light microscopy imaging, samples were transferred to PBS containing Hoechst 33258 (2 μg/mL, Invitrogen, cat no. H3570) and wheat germ agglutinin Alexa Fluor 647 Conjugate (Thermo Fisher Scientific, cat no. W32466). Overviews of the selected areas and individual cells were acquired using a Nikon spinning disc system consisting of a Yokogawa W2 and two Andor iXON888 cameras with NIS elements for image acquisition. The positions of the selected cells were recorded. A Nikon 100× 1.49 NA Apo-TIRF lens was used. The system was equipped with standard laser lines of 405, 488, 561, and 640 nm and the corresponding filter sets. For electron microscopy, samples were stained with 1% osmium tetroxide solution (Science Services, cat no. E19152) in PBS, followed by incubation with 2% uranyl acetate solution (Merck KgaA, Darmstadt, Germany) in water and subsequently dehydrated with a dehydration series ranging from 50% ethanol in water to 100% ethanol rotipuran (Roth, cat no. 9065.1). Over 2 days, the dehydrated samples were infiltrated with an EPON-ethanol series ranging from 50% EPON in ethanol to 100% EPON. A thin EPON layer was applied and polymerized overnight at 60°C. The samples were cut to the size of the preselected cells, and 50-nm-thick ultrathin sections were cut using a microtome equipped with a diamond knife and post-stained with 2% uranyl acetate solution in water. Electron microscopy was performed using a FEI Tecnai F20 electron microscope (Thermo Fisher Scientific), and images were captured with a side-mounted Olympus Veleta camera (Olympus Life Sciences). Image processing and analysis was performed in ImageJ/FIJI. Overlays were done in GIMP.

### Construction of expression plasmids.

All oligonucleotides used for plasmid cloning are given in Table S2. To generate pcDNA-MCP, the MCP ORF was subcloned into pcDNA3.1+ (Thermo Fisher Scientific) from HMCV BAC pHB5 by Red-mediated recombination (37). To this end, pcDNA3.1+ was cut with BamHI followed by treatment with calf intestine alkaline phosphatase (NEB, Ipswich, MA). The linearized vector was then used for PCR amplification employing primer pair MCP.for and MCP.rev, providing regions homologous to the 3′ or the 5′ end of the MCP ORF, respectively. Recombination-proficient GS1783 bacteria (43) harboring the HCMV BAC were transformed with the resulting PCR product, and bacterial clones containing pcDNA-MCP were selected on agar plates containing 100 μg/mL ampicillin. pmGFP was constructed employing a QuikChange site-directed mutagenesis protocol using the primers mGFP.for and mGFP.rev with pEGFP-C1 (TaKaRa Bio) as the PCR template. Following removal of template DNA with DpnI, ligation of the PCR amplicon by action of T4 ligase (NEB) and transformation of E. coli DH10B (NEB), clones harboring pmGFP were selected on agar plates containing 50 μg/mL kanamycin. pmGFP-SCP was constructed via Gibson Assembly (see above) using pmGFP cleaved with BglII as vector, and primer pair SCP.for and SCP.rev to amplify the SCP sequences from pHB5. Likewise, pmGFP-SCP-D1, -D2, and -D3 were obtained by Gibson Assembly utilizing the same vector and pHB5 as the PCR template. The primer combinations used were as follows: SCP-D1.for and SCP.rev for pmGFP-SCP-D1, SCP-D2.for and SCP.rev for pmGFP-SCP-D2, and SCP.for and SCP-D3.rev for pmGFP-SCP-D3. pmGFP-SCP-D4, -D5, and -D6 were also cloned via the QuikChange mutagenesis approach, with pmGFP-SCP as the PCR template and the following primer combinations: SCP-D4.for and SCP-D4.rev for pmGFP-SCP-D4, SCP-D5.for and SCP-D4.rev for pmGFP-SCP-D5, and SCP-D6.for and SCP-D6.rev for pmGFP-SCP-D6. The integrity of all plasmids was verified by restriction enzyme analysis and sequencing.

## References

[1] Mocarski ES, Shenk T, Griffiths PD, Pass RF. 2013. Cytomegaloviruses, p 1960–2014. *In* Knipe DM, Howley PM (ed), Fields virology. Lippincott Williams & Wilkins, Philadelphia, PA.

[2] Close WL, Anderson AN, Pellett PE. 2018. Betaherpesvirus virion assembly and egress. Adv Exp Med Biol 1045:167–207. doi:10.1007/978-981-10-7230-7_9. 29896668

[3] Borst EM, Mathys S, Wagner M, Muranyi W, Messerle M. 2001. Genetic evidence of an essential role for cytomegalovirus small capsid protein in viral growth. J Virol 75:1450–1458. doi:10.1128/JVI.75.3.1450-1458.2001. 11152518 PMC114051

[4] Desai P, DeLuca NA, Person S. 1998. Herpes simplex virus type 1 VP26 is not essential for replication in cell culture but influences production of infectious virus in the nervous system of infected mice. Virology 247:115–124. doi:10.1006/viro.1998.9230. 9683577

[5] Dai X, Gong D, Xiao Y, Wu T, Sun R, Zhou ZH. 2015. CryoEM and mutagenesis reveal that the smallest capsid protein cements and stabilizes Kaposi’s sarcoma-associated herpesvirus capsid. Proc Natl Acad Sci USA 112:E649–E656. doi:10.1073/pnas.1420317112. 25646489 PMC4343125

[6] Britt B. 2007. Maturation and egress. *In* Arvin A, Campadelli-Fiume G, Mocarski E, Moore PS, Roizman B, Whitely R, Yamanishi K (ed), Human herpesviruses: biology, therapy, and immunoprophylaxis. Cambridge University Press, Cambridge, United Kingdom. 21348071

[7] Gibson W. 2008. Structure and Formation of the Cytomegalovirus Virion, p 187–204. *In* Shenk TE, Stinski MF (ed), Human cytomegalovirus. Springer Berlin Heidelberg, Berlin, Germany. 10.1007/978-3-540-77349-8_11 18637507

[8] Gibson W, Bogner E. 2013. Morphogenesis of the cytomegalovirus virion and subviral particles, p 230–246. *In* Reddehase MJ, Lemmermann NAW (ed), Cytomegaloviruses. Caister Academic Press, Norfolk, United Kingdom.

[9] Tandon R, Mocarski ES, Conway JF. 2015. The A, B, Cs of herpesvirus capsids. Viruses 7:899–914. doi:10.3390/v7030899. 25730559 PMC4379554

[10] Tandon R, Mocarski ES. 2012. Viral and host control of cytomegalovirus maturation. Trends Microbiol 20:392–401. doi:10.1016/j.tim.2012.04.008. 22633075 PMC3408842

[11] DeRussy BM, Tandon R. 2015. Human cytomegalovirus pUL93 is required for viral genome cleavage and packaging. J Virol 89:12221–12225. doi:10.1128/JVI.02382-15. 26401033 PMC4645300

[12] Borst EM, Bauerfeind R, Binz A, Stephan TM, Neuber S, Wagner K, Steinbrück L, Sodeik B, Lenac Roviš T, Jonjić S, Messerle M. 2016. The essential human cytomegalovirus proteins pUL77 and pUL93 are structural components necessary for viral genome encapsidation. J Virol 90:5860–5875. doi:10.1128/JVI.00384-16. 27009952 PMC4907240

[13] McNab AR, Desai P, Person S, Roof LL, Thomsen DR, Newcomb WW, Brown JC, Homa FL. 1998. The product of the herpes simplex virus type 1 UL25 gene is required for encapsidation but not for cleavage of replicated viral DNA. J Virol 72:1060–1070. doi:10.1128/JVI.72.2.1060-1070.1998. 9445000 PMC124578

[14] Stow ND. 2001. Packaging of genomic and amplicon DNA by the herpes simplex virus type 1 UL25-null mutant KUL25NS. J Virol 75:10755–10765. doi:10.1128/JVI.75.22.10755-10765.2001. 11602717 PMC114657

[15] Klupp BG, Granzow H, Keil GM, Mettenleiter TC. 2006. The capsid-associated UL25 protein of the alphaherpesvirus pseudorabies virus is nonessential for cleavage and encapsidation of genomic DNA but is required for nuclear egress of capsids. J Virol 80:6235–6246. doi:10.1128/JVI.02662-05. 16775311 PMC1488961

[16] Mettenleiter TC, Klupp BG, Granzow H. 2009. Herpesvirus assembly: an update. Virus Res 143:222–234. doi:10.1016/j.virusres.2009.03.018. 19651457

[17] Heming JD, Conway JF, Homa FL. 2017. Herpesvirus capsid assembly and DNA packaging. Adv Anat Embryol Cell Biol 223:119–142. doi:10.1007/978-3-319-53168-7_6. 28528442 PMC5548147

[18] McElwee M, Vijayakrishnan S, Rixon F, Bhella D. 2018. Structure of the herpes simplex virus portal-vertex. PLoS Biol 16:e2006191. doi:10.1371/journal.pbio.2006191. 29924793 PMC6028144

[19] Liu Y, Jih J, Dai X, Bi G, Zhou ZH. 2019. Cryo-EM structures of herpes simplex virus type 1 portal vertex and packaged genome. Nature 570:257–261. doi:10.1038/s41586-019-1248-6. 31142842 PMC6732574

[20] Li Z, Pang J, Dong L, Yu X. 2021. Structural basis for genome packaging, retention, and ejection in human cytomegalovirus. Nat Commun 12:4538. doi:10.1038/s41467-021-24820-3. 34315863 PMC8316551

[21] Naniima P, Naimo E, Koch S, Curth U, Alkharsah KR, Ströh LJ, Binz A, Beneke J, Vollmer B, Böning H, Borst EM, Desai P, Bohne J, Messerle M, Bauerfeind R, Legrand P, Sodeik B, Schulz TF, Krey T. 2021. Assembly of infectious Kaposi’s sarcoma-associated herpesvirus progeny requires formation of a pORF19 pentamer. PLoS Biol 19:e3001423. doi:10.1371/journal.pbio.3001423. 34735435 PMC8568140

[22] Bhella D, Rixon FJ, Dargan DJ. 2000. Cryomicroscopy of human cytomegalovirus virions reveals more densely packed genomic DNA than in herpes simplex virus type 1. J Mol Biol 295:155–161. doi:10.1006/jmbi.1999.3344. 10623515

[23] Yu X, Jih J, Jiang J, Zhou ZH. 2017. Atomic structure of the human cytomegalovirus capsid with its securing tegument layer of pp150. Science 356:eaam6892. doi:10.1126/science.aam6892. 28663444 PMC5715728

[24] Yu X, Shah S, Lee M, Dai W, Lo P, Britt W, Zhu H, Liu F, Zhou ZH. 2011. Biochemical and structural characterization of the capsid-bound tegument proteins of human cytomegalovirus. J Struct Biol 174:451–460. doi:10.1016/j.jsb.2011.03.006. 21459145 PMC3684277

[25] Liu W, Dai X, Jih J, Chan K, Trang P, Yu X, Balogun R, Mei Y, Liu F, Zhou ZH. 2019. Atomic structures and deletion mutant reveal different capsid-binding patterns and functional significance of tegument protein pp150 in murine and human cytomegaloviruses with implications for therapeutic development. PLoS Pathog 15:e1007615. doi:10.1371/journal.ppat.1007615. 30779794 PMC6396938

[26] Tandon R, Mocarski ES. 2008. Control of cytoplasmic maturation events by cytomegalovirus tegument protein pp150. J Virol 82:9433–9444. doi:10.1128/JVI.00533-08. 18653449 PMC2546967

[27] Dai X, Yu X, Gong H, Jiang X, Abenes G, Liu H, Shivakoti S, Britt WJ, Zhu H, Liu F, Zhou ZH. 2013. The smallest capsid protein mediates binding of the essential tegument protein pp150 to stabilize DNA-containing capsids in human cytomegalovirus. PLoS Pathog 9:e1003525. doi:10.1371/journal.ppat.1003525. 23966856 PMC3744435

[28] Britt B. 2007. Maturation and egress. *In* Arvin A, Campadelli-Fiume G, Mocarski E, Moore PS, Roizman B, Whitley R, Yamanishi K (ed), Human herpesviruses: biology, therapy, and immunoprophylaxis. Cambridge University Press, Cambridge, United Kingdom. 21348071

[29] Elbasani E, Gabaev I, Steinbrück L, Messerle M, Borst EM. 2014. Analysis of essential viral gene functions after highly efficient adenofection of cells with cloned human cytomegalovirus genomes. Viruses 6:354–370. doi:10.3390/v6010354. 24452007 PMC3917448

[30] Borst EM, Wagner K, Binz A, Sodeik B, Messerle M. 2008. The essential human cytomegalovirus gene UL52 is required for cleavage-packaging of the viral genome. J Virol 82:2065–2078. doi:10.1128/JVI.01967-07. 18077717 PMC2258901

[31] Couté Y, Kraut A, Zimmermann C, Büscher N, Hesse A, Bruley C, De Andrea M, Wangen C, Hahn F, Marschall M, Plachter B. 2020. Mass spectrometry-based characterization of the virion proteome, phosphoproteome, and associated kinase activity of human cytomegalovirus. Microorganisms 8:820. doi:10.3390/microorganisms8060820. PMC7357008 32486127

[32] Tandon R, Mocarski ES. 2011. Cytomegalovirus pUL96 is critical for the stability of pp150-associated nucleocapsids. J Virol 85:7129–7141. doi:10.1128/JVI.02549-10. 21593167 PMC3126555

[33] Lai L, Britt WJ. 2003. The interaction between the major capsid protein and the smallest capsid protein of human cytomegalovirus is dependent on two linear sequences in the smallest capsid protein. J Virol 77:2730–2735. doi:10.1128/jvi.77.4.2730-2735.2003. 12552013 PMC141075

[34] Nagel C, Döhner K, Binz A, Bauerfeind R, Sodeik B. 2012. Improper tagging of the non-essential small capsid protein VP26 impairs nuclear capsid egress of herpes simplex virus. PLoS One 7:e44177. doi:10.1371/journal.pone.0044177. 22952920 PMC3432071

[35] Zimmermann C, Krämer N, Krauter S, Strand D, Sehn E, Wolfrum U, Freiwald A, Butter F, Plachter B. 2021. Autophagy interferes with human cytomegalovirus genome replication, morphogenesis, and progeny release. Autophagy 17:779–795. doi:10.1080/15548627.2020.1732686. 32079454 PMC8032242

[36] Borst EM, Hahn G, Koszinowski UH, Messerle M. 1999. Cloning of the human cytomegalovirus (HCMV) genome as an infectious bacterial artificial chromosome in *Escherichia coli*: a new approach for construction of HCMV mutants. J Virol 73:8320–8329. doi:10.1128/JVI.73.10.8320-8329.1999. 10482582 PMC112849

[37] Tischer BK, von Einem J, Kaufer B, Osterrieder N. 2006. Two-step Red-mediated recombination for versatile high-efficiency markerless DNA manipulation in *Escherichia coli*. Biotechniques 40:191–197. doi:10.2144/000112096. 16526409

[38] Hammer Q, Rückert T, Borst EM, Dunst J, Haubner A, Durek P, Heinrich F, Gasparoni G, Babic M, Tomic A, Pietra G, Nienen M, Blau IW, Hofmann J, Na I, Prinz I, Koenecke C, Hemmati P, Babel N, Arnold R, Walter J, Thurley K, Mashreghi M, Messerle M, Romagnani C. 2018. Peptide-specific recognition of human cytomegalovirus strains controls adaptive natural killer cells. Nat Immunol 19:453–463. doi:10.1038/s41590-018-0082-6. 29632329

[39] Glass M, Busche A, Wagner K, Messerle M, Borst EM. 2009. Conditional and reversible disruption of essential herpesvirus proteins. Nat Methods 6:577–579. doi:10.1038/nmeth.1346. 19578384

[40] Borst E, Messerle M. 2005. Analysis of human cytomegalovirus oriLyt sequence requirements in the context of the viral genome. J Virol 79:3615–3626. doi:10.1128/JVI.79.6.3615-3626.2005. 15731256 PMC1075693

[41] Borst EM, Kleine-Albers J, Gabaev I, Babic M, Wagner K, Binz A, Degenhardt I, Kalesse M, Jonjic S, Bauerfeind R, Messerle M. 2013. The human cytomegalovirus UL51 protein is essential for viral genome cleavage-packaging and interacts with the terminase subunits pUL56 and pUL89. J Virol 87:1720–1732. doi:10.1128/JVI.01955-12. 23175377 PMC3554196

[42] Vornhagen R, Plachter B, Hinderer W, The TH, Van Zanten J, Matter L, Schmidt CA, Sonneborn HH, Jahn G. 1994. Early serodiagnosis of acute human cytomegalovirus infection by enzyme-linked immunosorbent assay using recombinant antigens. J Clin Microbiol 32:981–986. doi:10.1128/jcm.32.4.981-986.1994. 8027354 PMC267166

[43] Tischer BK, Smith GA, Osterrieder N. 2010. *En passant* mutagenesis: a two step markerless Red recombination system. Methods Mol Biol 634:421–430. doi:10.1007/978-1-60761-652-8_30. 20677001

[44] Neuber S, Wagner K, Goldner T, Lischka P, Steinbrueck L, Messerle M, Borst EM. 2017. Mutual Interplay between the human cytomegalovirus terminase subunits pUL51, pUL56, and pUL89 promotes terminase complex formation. J Virol 91:e02384-16. doi:10.1128/JVI.02384-16. 28356534 PMC5446633

