# Supplementary material for: A Unique Role of the Human Cytomegalovirus Small Capsid Protein in Capsid Assembly
Source: mBio. 2022 Sep 6;13(5):e01007-22. doi: 10.1128/mbio.01007-22 (PMC9600257; doi:10.1128/mbio.01007-22)
Supplement: TABLE S1 [file mbio.01007-22-s0005.docx]

**Table S1: HCMV BACs employed in this study**

| **Name** | **Features** | **References** |
| --- | --- | --- |
| HB5-UL77gfp | AD169 BAC genome pHB5 expressing a pUL77-mGFP fusion protein  (HB5-UL77-mGFP-3) | (1, 2) |
| HB5-UL77gfp-ΔMCP | MCP ORF disrupted in HB5-UL77-mGFP-3 | (2) |
| HB5-ΔUL77 | AD169 BAC genome pHB5 carrying a large deletion in the UL77 ORF | this study |
| HB5-UL77gfp-ΔUL93s | UL93 ORF disrupted in HB5-UL77-mGFP-3-ΔUL93 | this study |
| HB5-UL77gfp-Δpp150c | UL32 ORF deleted in HB5-UL77-mGFP-3 | this study |
| HB5-UL77gfp-ΔSCP | Deletion in ORF UL48.5 in HB5-UL77-mGFP-3 | this study |
| HB5-ΔSCP | AD169 BAC genome pHB5 harboring a deletion in ORF UL48.5 | (3) |
| HB5-SCPgfp | AD169 BAC genome pHB5 with EGFP inserted between aa 8 and 9 (P/T) of SCP | (3) |
| HB5-SCP-mRFP | AD169 BAC genome pHB5 with mRFP inserted between aa 4 and 5 (T/A) of SCP | this study |

Individual ORFs were either completely or partially deleted (depending on their vicinity to or overlap with neighboring ORFs), in some mutants concomitantly with the introduction of stop codons. HB5-ΔUL77 was constructed analogous to the previously published HG-ΔUL77 (2), except that the HB5 BAC does not express EGFP. HB5-UL77gfp-ΔUL93s differs from HB5-UL77-mGFP-3-ΔUL93 (expressing a C-terminally truncated pUL93 version (2)) in that two stop codons were inserted after the alanine codon at position 16 of the UL93 ORF, thereby completely abrogating UL93 expression (data not shown).

**References**

1. Borst EM, Hahn G, Koszinowski UH, Messerle M. 1999. Cloning of the human cytomegalovirus (HCMV) genome as an infectious bacterial artificial chromosome in Escherichia coli: a new approach for construction of HCMV mutants. J Virol 73:8320-8329. 10.1128/JVI.73.10.8320-8329.1999.

2. Borst EM, Bauerfeind R, Binz A, Stephan TM, Neuber S, Wagner K, Steinbrück L, Sodeik B, Lenac Roviš T, Jonjić S, Messerle M. 2016. The Essential Human Cytomegalovirus Proteins pUL77 and pUL93 Are Structural Components Necessary for Viral Genome Encapsidation. J Virol 90:5860-5875. 10.1128/JVI.00384-16.

3. Borst EM, Mathys S, Wagner M, Muranyi W, Messerle M. 2001. Genetic evidence of an essential role for cytomegalovirus small capsid protein in viral growth. J Virol 75:1450-1458. 10.1128/JVI.75.3.1450-1458.2001.

stylefix
