# Supplementary figures and images for: A Unique Role of the Human Cytomegalovirus Small Capsid Protein in Capsid Assembly
Source: mBio. 2022 Sep 6;13(5):e01007-22. doi: 10.1128/mbio.01007-22 (PMC9600257; doi:10.1128/mbio.01007-22)

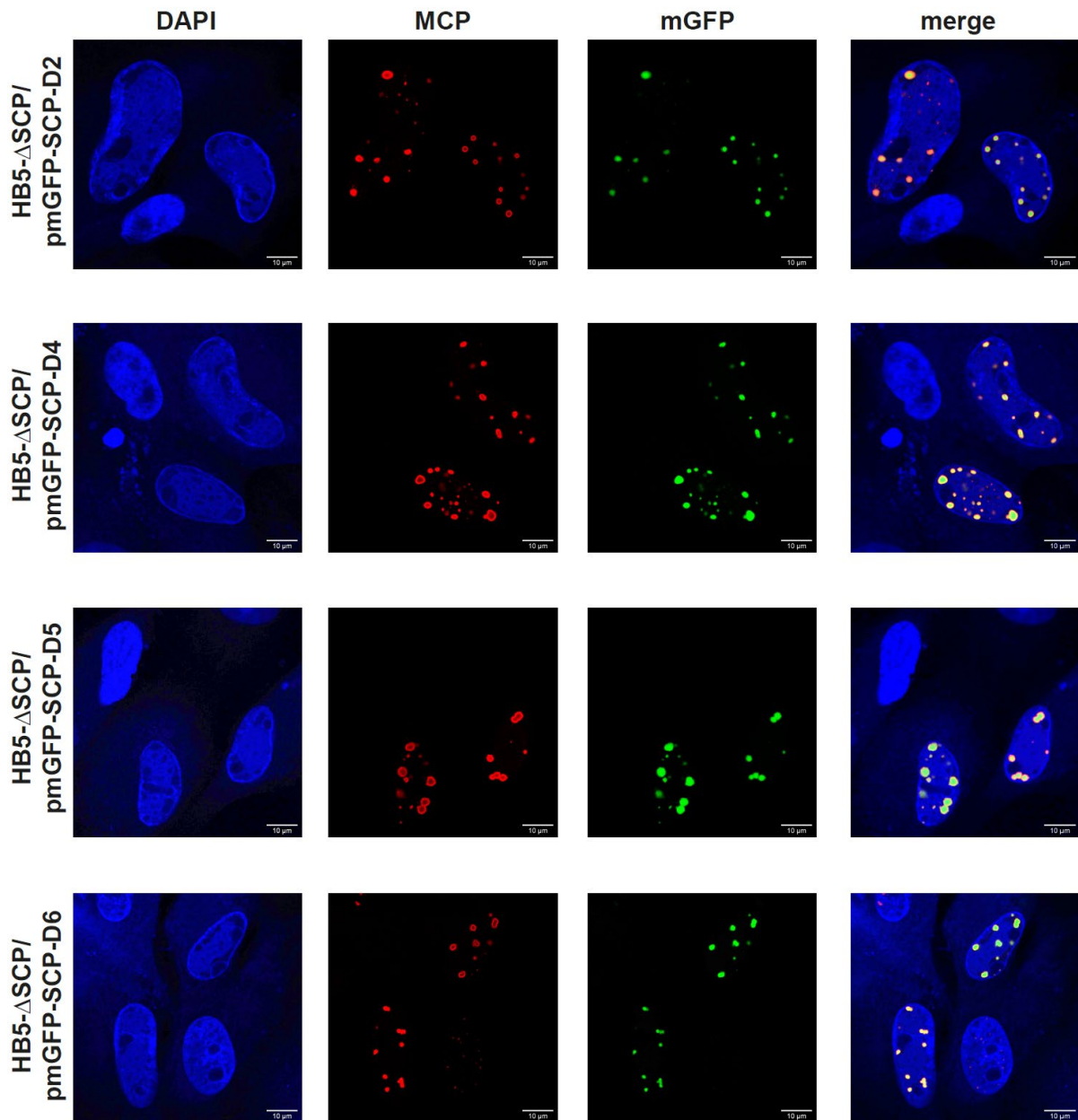

Supplement: FIG S1 [file mbio.01007-22-s0001.pdf]

Light  
microscopy

Overview

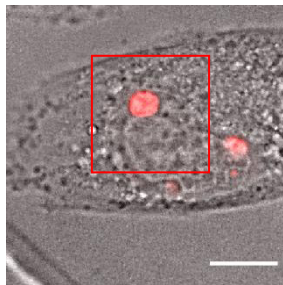

Zoom

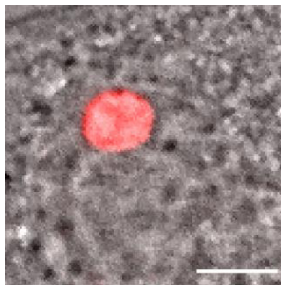

CLEM

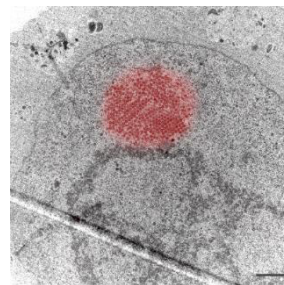

Electron  
microscopy

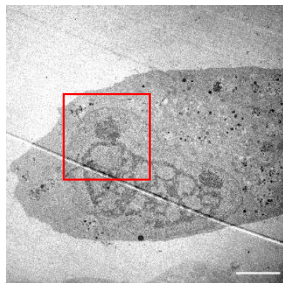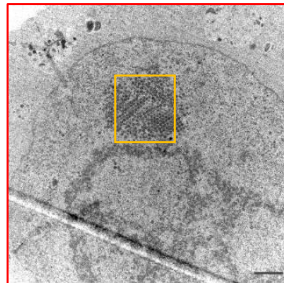

Detail

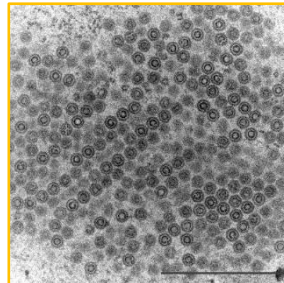

Supplement: FIG S3 [file mbio.01007-22-s0003.pdf]
