# Supplementary material for: A Unique Role of the Human Cytomegalovirus Small Capsid Protein in Capsid Assembly
Source: mBio. 2022 Sep 6;13(5):e01007-22. doi: 10.1128/mbio.01007-22 (PMC9600257; doi:10.1128/mbio.01007-22)
Supplement: TABLE S2 [file mbio.01007-22-s0006.docx]

**Table S2: Oligonucleotides used in this study.**

| **Name** | **Sequence (5’ > 3’)** |
| --- | --- |
| UL93-KO-stop.for | gtattcggatctggcgtttgaagcgcggttcgcttagtaaagcaattg  cctctacacttgacgcatcgtggccggatc |
| UL93-KO-stop.rev | ctcctggtcgagcaccaagtgtagaggcaattgctttactaagcgaac  cgcgcttgtgaccacgtcgtggaatg |
| D-pp150.for | tggtttcattaaaaagtacgtctgcgtgtgtgtttcttaagctggcttttctc  cagaaccacgcatcgtggccggatc |
| D-pp150.rev | ccggggcgacgacgcttccgggttctggagaaaagccagcttaagaa  acacacacgcagagtgaccacgtcgtggaatg |
| SCP-KO.for | ccgggtgggtggcctctgatatctcggtcggcagctccaactagttacct  attatttttcgtcccgccaaggatgacgacgataag |
| SCP-KO.rev | cgcgccgggacccacggtggccaacaagcgggacgaaaaataatag  gtaactagttggagctgccgagccagtgttacaaccaatt |
| SCP-mRFP.for | gtttttcgtcccgcttgttggccaccgtgggtcccggcgcggcgccggtggagtggcgg |
| SCP-mRFP.rev | tcttcgtcctccccccacggcctgccccatgtctaacaccgcctcctccgaggacgtca |
| MCP.for | gaaatcatcccgctgcagcaatccatgttatttaactcgtgagatccactagtccagtgtggtg |
| MCP.rev | gcctactttaggcaggagctcgagcgccgaccagttctccatggtggaagcttaagtttaaacgctagcc |
| mGFP.for | P~cctgagcacccagtccaagctgagcaaagaccccaac |
| mGFP.rev | P~gttggggtctttgctcagcttggactgggtgctcagg |
| SCP.for | tacaagtccggactcagatctggagcctcgtctaacaccgcgccgggacc |
| SCP.rev | cgaagcttgagctcgagatcttcagcgccgggtgcgcga |
| SCP-D1.for | tacaagtccggactcagatctggagcctcggccaacaagcgggacgaaaaacaccg |
| SCP-D2.for | tacaagtccggactcagatctggagcctcggagctgccgaccgagatatcagaggc |
| SCP-D3.rev | cgaagcttgagctcgagatcttcaattaaacaggctggacatgcgc |
| SCP-D4.for | P~ acccacccggtgttggcca |
| SCP-D4.rev | P~ cgaggctccagatctgagtcc |
| SCP-D5.for | P~ aatgacaagtgcgcctttaagc |
| SCP-D6.for | P~ gacaagtgcgcctttaagctgg |
| SCP-D6.rev | P~ gtgggtggcctctgatatctcg |
| UL32-BCE2.for | tcacggatccgcatgcgatgtcgtgtcccccgc |
| UL32-BCE2.rev | ccaagctcagctaattaagcttctattcctccgtgttcttaatcttctcg |
| MCP-  qPCR.for | cgttcatagggaagacgcga |
| MCP-qPCR.rev | tagcctacgtaaaccgcgtg |

P~: oligonucleotides phosphorylated at the 5’-end.
